# Pharmacogenetic Implications for Antidepressant Therapy in Major Depression: A Systematic Review Covering 2019–2024

**DOI:** 10.3390/jcm14145102

**Published:** 2025-07-18

**Authors:** Anna Fornaguera, Marta Miarons

**Affiliations:** 1 Faculty of Pharmacy, Barcelona University, 08001 Barcelona, Spain; 2 Pharmacy Service, Consorci Hospitalari de Vic, 08500 Barcelona, Spain

**Keywords:** pharmacogenetics, antidepressants, major depressive disorder, late-life depression

## Abstract

**Background/Objectives**: Major depressive disorder (MDD), including late-onset forms, is a prevalent and disabling condition. Despite multiple pharmacological treatment options, over half of patients fail to achieve full remission. This systematic review aims to assess current evidence on the influence of pharmacogenetic factors on antidepressant response and safety, with a focus on patients with major and late-life depression. **Methods**: We conducted a systematic review following PRISMA guidelines (PROSPERO: CRD42020212345). Studies published in the past five years involving adult patients with MDD or late-onset depression and pharmacogenetic data were included. **Results**: From 793 abstracts screened, 29 studies with 39,975 participants were included. CYP2C19 and CYP2D6 were the most frequently analyzed genes (41% and 17% of studies, respectively). Poor metabolizers for CYP2C19 showed higher plasma levels of SSRIs, leading to increased adverse effects. In contrast, ultrarapid metabolizers had significantly lower response rates. Variants in SLC6A4 and other genes (e.g., HTR2A, ABCB1) were also associated with treatment outcomes. Combinatorial pharmacogenetic testing showed superior predictive value compared to single-gene approaches. **Conclusions**: Genetic variants in CYP2C19, CYP2D6, and SLC6A4 may affect the efficacy and tolerability of antidepressant therapy. Integrating this information into clinical practice may allow more personalized prescribing and improved outcomes.

## 1. Introduction

Major depressive disorder (MDD), including late-onset forms, is a chronic and disabling psychiatric condition that, according to the World Health Organization (WHO), affects approximately 280 million people worldwide [1]. This disorder represents a major public health concern due to the large proportion of the population it impacts. It is characterized by a persistently depressed mood, loss of pleasure, or disinterest in activities for extended periods of time [1].

Among the clinical subtypes of MDD is late-onset depression, which typically occurs after the age of 60 and is associated with neurobiological changes, physical illness, cognitive decline, or losses related to age-related losses [2]. Recent evidence, such as the national survey by You et al. [3], highlights a robust inverse association between cardiovascular health and depressive symptoms, identifying modifiable lifestyle factors such as sleep and physical activity as key intervention targets. This multidimensional approach underscores the biological and behavioral mechanisms connecting health behaviors with depression, emphasizing that lifestyle interventions can be as critical as pharmacotherapy in managing depressive disorders.

Although several therapeutic options exist, up to 57.9% of patients with depression do not achieve full remission with standard antidepressant treatment [4]. This highlights the limitations of current pharmacological strategies and the need to identify factors that can better predict treatment response and inform clinical decision-making. Currently, the most widely used first-line antidepressants are selective serotonin reuptake inhibitors (SSRIs), such as citalopram and escitalopram, followed by serotonin-norepinephrine reuptake inhibitors (SNRIs) like venlafaxine, tricyclic antidepressants (TCAs), and other agents such as bupropion and ketamine [5].

Despite their potential efficacy, treatment response and remission rates remain unsatisfactory due to the lack of reliable predictors of outcome. Interindividual genetic differences that affect how patients metabolize and respond to medications may partly explain this variability. These genetic variations can influence both the pharmacokinetics (absorption, distribution, metabolism, and excretion) and pharmacodynamics of antidepressants [6].

The main enzymes involved in the metabolism of antidepressants belong to the cytochrome P450 family, particularly CYP2C19 and CYP2D6. Genetic polymorphisms in these genes define metabolizer phenotypes ranging from poor to ultrarapid, with direct implications for drug efficacy and safety [7]. In fact, studies have shown that only around 17–25% of the population has a normal metabolic phenotype for both CYP2D6 and CYP2C19, although this figure varies considerably depending on ancestry [8]. This underscores the significant interindividual variability in antidepressant response.

Beyond these, other genes related to serotonin transporters (e.g., *SLC6A4*) and receptors (e.g., *HTR2A*) may also influence therapeutic response and adverse effects. Genetic variants in the promoter region of *SLC6A4*, known as 5-HTTLPR, affect synaptic serotonin availability and indirectly impact treatment efficacy and remission rates [9,10].

In addition, combinatorial pharmacogenetic tests, which assess multiple pharmacokinetic and pharmacodynamic factors simultaneously, have demonstrated greater predictive value than single-gene analyses, offering a more accurate prediction of treatment response and tolerability [11].

In this context, pharmacogenetics emerges as a promising tool to improve therapeutic management of depression, particularly in this population subgroup. Studying individual genetic variations may help predict treatment response, minimize adverse effects, and guide personalized drug selection. Despite growing research in this field, consensus is still lacking, justifying the need for a systematic review that provides a comprehensive and up-to-date overview of the current scientific evidence regarding genetic factors influencing antidepressant response in patients with late-onset or major depression.

## 2. Materials and Methods

### 2.1. Literature Search Strategy

Researchers identified studies assessing the pharmacogenetic implications for antidepressant pharmacotherapy in late-life depression through a systematic review of the literature following the 2020 Preferred Reporting Items for Systematic Reviews and Meta-Analyses (PRISMA) guidelines [12]. The PRISMA checklist was followed to ensure transparent and complete reporting of the review process.

The search was carried out using three databases: PubMed, Scifinder, and Cochrane. To narrow the search, the researches applied the following filters: articles published within the last five years, human studies, and publications in English or Spanish, and carefully selected Medical Subject Headings (MeSH) terms and database-specific keywords to ensure comprehensive coverage of topics related to pharmacogenetics, antidepressant pharmacotherapy, and major or late-onset depression. These terms were used to conduct the search systematically across all databases to retrieve all relevant studies. Keywords included general terms such as “pharmacogenetics”, “antidepressants”, “late-life depression”, and “genetic variability”, as well as specific MeSH or controlled terms detailed in Table 1.

The authors limited the search to the last five years to ensure the inclusion of up-to-date evidence that reflects recent advances in pharmacogenetic research, clinical practice guidelines, and methodological standards.

This systematic review was registered in PROSPERO (registration number: CRD42020212345). This comprehensive search strategy allowed the researchers to identify relevant literature, and carry out rigorous screening based on predefined eligibility criteria.

### 2.2. Selection Criteria

Two independent reviewers screened article abstracts from the search results to assess their eligibility based on predefined inclusion and exclusion criteria (Table 2).

Articles were included when both reviewers agreed on eligibility. In case of disagreement, each reviewer presented arguments based on the selection criteria until consensus was reached.

Full-text versions of all potentially eligible articles (except three that could not be retrieved) were obtained and re-assessed for inclusion by both reviewers.

### 2.3. Data Extraction

The two reviewers developed and approved a customized data extraction form to ensure consistency and data quality. The form included the following items: first author, year of publication, study type (clinical trial, prospective or retrospective observational), level of evidence based on the GRADE system, type of antidepressant used, and characteristics of the study population.

Additionally, it included information on the analysis of specific genetic polymorphisms associated with antidepressant response, as well as clinical outcomes assessed (e.g., symptom severity, treatment response, adverse effects).

The reviewers documented the main findings from each study, focusing on the relationship between genetic variation and antidepressant response, and the impact of these factors on treatment efficacy and safety.

A qualitative heatmap was constructed to visually summarise the evidence for associations between pharmacogenetic polymorphisms and treatment outcomes (efficacy, safety, and drug concentration).

### 2.4. Level of Evidence and Risk of Bias

The quality of the included studies was assessed using the GRADE methodology [13], which classifies evidence into four levels: very low, low, moderate, and high. The classification was based on:

1. Study Design: Randomized controlled trials (RCTs) were considered high quality; observational studies were deemed lower quality.
2. Consistency: Quality increased when findings were consistent across studies.
3. Precision: Higher weight was given to studies with narrow confidence intervals and large sample sizes.
4. Risk of Bias: Studies employing randomization and blinding were rated higher.
5. Directness: Evidence directly applicable to the research question was considered higher quality.

This system facilitated the assessment of confidence in the review’s conclusions and guided clinical recommendations.

The risk of bias of the eligible studies was assessed through discussion and consensus among the investigators, with particular attention to the use of objective evaluation criteria and the representativeness of the sample. The risk of bias of the included observational studies was assessed according to the Newcastle-Ottawa Scale (NOS) recommendation. Eight items were selected for study inclusion, including patient selection, comparability between groups, and exposure factors. Studies with NOS scores of 0–3, 4–6, and 7–9 were considered to have high, moderate, and low risk of bias, respectively [14,15].

### 2.5. Subgroup Analysis

Subgroup analyses based on age, antidepressant type, or specific genetic polymorphisms could not be conducted due to the high heterogeneity of the included studies.

## 3. Results

### 3.1. Study Selection

**Figure 1:**
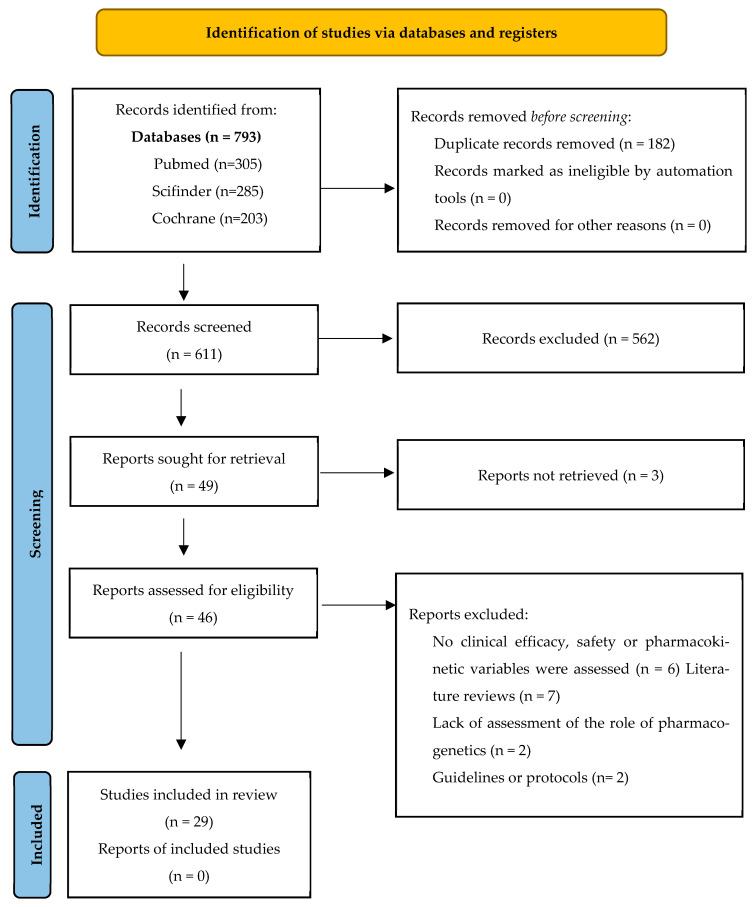
PRISMA 2020 flow diagram illustrating the seletion process for studies included in the systematic review.

The selection process is detailed in the adapted PRISMA flow diagram (Figure 1). The completed PRISMA 2020 checklist is provided as Table S1. 

From the initial bibliographic search, 793 records were identified from PubMed, Scifinder, and Cochrane. After removing duplicates, 611 unique articles remained.

Following the initial screening of titles and abstracts, 562 articles were excluded for not meeting the predefined inclusion criteria. Thus, 49 articles were selected for full-text review. However, three full texts could not be retrieved, leaving 46 articles eligible for further assessment.

Upon full-text evaluation, 17 articles were excluded for not meeting the eligibility criteria. Ultimately, 29 articles were included in the systematic review. There was full agreement between the two reviewers on the inclusion and exclusion decisions.

### 3.2. Study Types, Quality and Risk of Bias

As shown in Table S2, the 29 studies included in the review were predominantly observational in design: 15 were prospective (51.7%) [16,17,18,19,20,21,22,23,24,25,26,27,28,29,30] and 13 were retrospective (44.83%) [31,32,33,34,35,36,37,38,39,40,41,42,43]. Only one study was a randomized clinical trial (3.45%) [44].

The quality of evidence was assessed using the GRADE approach. Based on this system, the studies were classified as follows: low quality (N = 8, 27.5%) [16,17,18,24,31,32,33,34], moderate quality (N = 20, 68.96%) [19,20,21,22,23,24,26,27,28,29,30,35,36,37,38,39,40,41,42,43], and high quality (N = 1, 3.45%) [44]. No studies were rated as very low quality.

The risk of bias, assessed using the NOS, showed that most studies were of moderate methodological quality overall (Table S3). Common limitations included insufficient adjustment for potential confounders and the absence of blinding in outcome assessment, both of which could introduce bias.

### 3.3. Participants

The characteristics of the 29 included studies are summarized in Table S2, encompassing a total of 39,975 participants. However, only four studies (13.79%) included more than 500 participants [20,38,40,41].

Pharmacogenetic implications were analyzed across diverse patient populations, often classified by ethnicity given its potential influence on treatment outcomes. Study populations included: Caucasian (N = 11, 37.93%) [16,18,24,25,26,27,28,31,35,36,37,38,39], East Asian (N = 3, 10.34%) [14,17,33], and unspecified ethnicity (N = 15, 51.72%) [19,21,22,23,29,30,31,32,33,34,38,40,41,42,43,44].

Participants spanned a wide age range, covering both major and late-onset depression subtypes. Late-onset depression, typically defined as onset after 60 years, was explicitly reported in only one study (3.44%) [41]. The remaining 28 studies (96.55%) focused on major depressive disorder, generally with a mean onset age of around 45 years [16,17,18,19,20,21,22,23,24,25,26,27,28,29,30,31,32,33,34,35,36,37,38,39,40,42,43,44].

### 3.4. Antidepressant Treatments

Analysis of antidepressant treatments revealed a broad range of pharmacological strategies used across the included studies. These were categorized into four main groups: SSRIs, serotonin-norepinephrine reuptake inhibitors (SNRIs), TCAs, and other antidepressants with diverse mechanisms of action.

SSRIs were the most frequently prescribed, reported in 24 studies (82.75%) [16,17,18,20,21,22,23,24,25,26,27,28,30,31,33,34,35,36,37,38,40,41,42,43]. SNRIs were reported in 12 studies (41.37%) [16,19,20,25,26,27,28,31,35,36,37,38]. Other commonly used agents included mirtazapine, bupropion, and intravenous ketamine.

### 3.5. Pharmacogenetic Determinations

A range of genetic variants and polymorphisms have been identified to determine their potential influence on clinical outcomes of antidepressant treatment, including therapeutic response (symptom improvement), remission (complete resolution of symptoms), as well as drug metabolism and safety.

**Figure 2:**
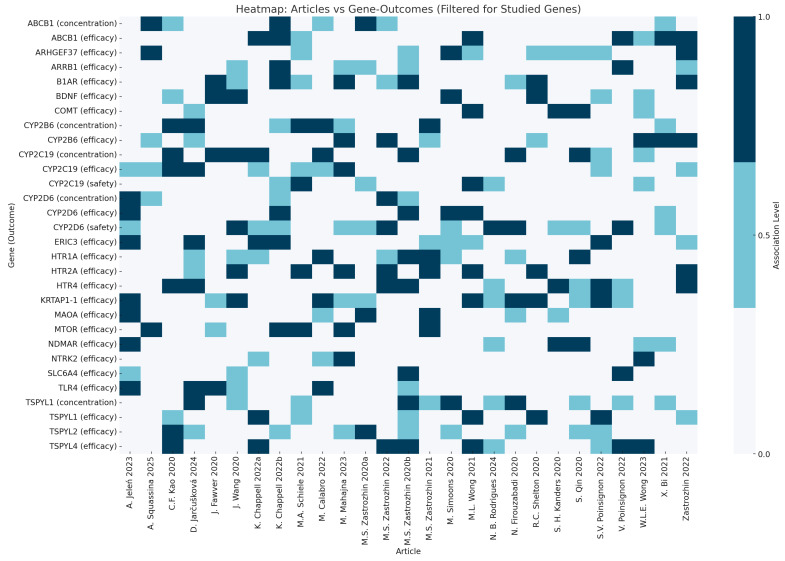
Heatmap showing the association between pharmacogenetic polymorphisms and treatment outcomes (efficacy, safety, or drug concentration) across different studies [16,17,18,19,20,21,22,23,24,25,26,27,28,29,30,31,32,33,34,35,36,37,38,39,40,41,42,43,44]. Each row represents a published study, identified by the first author and, when necessary, the study reference (in parentheses). Each column corresponds to a gene, annotated with the relevant outcome (e.g., efficacy, safety, or concentration). The color scale indicates whether an association was found: dark blue (significant association), lighter blue (no association), and very light blue (not studied).

To provide a comprehensive visual overview, a qualitative heatmap was developed (Figure 2) to illustrate the evidence across studies for associations between individual pharmacogenetic variants and treatment outcomes. This heatmap allows for the identification of genes most consistently associated with efficacy, safety, or drug concentration, as well as areas where evidence is limited or inconsistent.

The most commonly studied variants were those in CYP2C19 (N = 12, 41.37%) [16,21,24,30,31,33,34,35,38,40,42,43], CYP2D6 (N = 5, 17.24%) [19,24,29,31,33], and SLC6A4 (N = 4, 13.79%) [20,24,25,42]. Less frequently examined genes included HTR2A (N = 2, 6.9%) [20,36], CYP2B6 (N = 2, 6.9%) [20,36], CYP3A4 (N = 2, 6.9%) [33,34], and combinatorial pharmacogenetic tests, assessed in 2 studies (6.9%). Additional variants were analyzed sporadically, including polymorphisms in ARHGEF37, TLR4, G1165C, BDNF, CYP2C9, CYP1A2, TSPYL1, TSPYL2, TSPYL4, ABCB1, MAOA, ARRB1, COMT, ERICH3, HTR4, NTRK2, MTOR, NDMAR, and KRTAP1-1.

#### 3.5.1. CYP2C19

Variants in the CYP2C19 gene were the most frequently analyzed in this review, given their role in the metabolism of many antidepressants [16,21,24,30,31,33,34,35,38,40,42,43].

These variants are categorized based on metabolizer phenotype (Table 3), which has clear implications for both drug efficacy and safety.

Among the most studied variants are rs4244285 (CYP2C19*2) and rs12248560 (CYP2C19*17). The *2 allele (681G>A) is associated with reduced enzyme activity, resulting in higher drug concentrations and potentially increased side effects and reduced tolerability. Conversely, the *17 allele (C>T) leads to increased enzymatic activity. Individuals homozygous for this variant (*17/*17) are considered ultrarapid metabolizers, which may cause lower plasma concentrations and reduced therapeutic response.

Across the reviewed studies, CYP2C19 ultrarapid metabolizers (*17) were consistently associated with lower treatment response and higher rates of therapeutic failure compared to normal (*1/*1) or poor (*2/*2) metabolizers.

In contrast, poor metabolizers often exhibited higher plasma concentrations due to reduced enzymatic activity, leading to an increased risk of adverse drug reactions and a greater likelihood of treatment discontinuation or dose adjustments.

Additionally, the rs3828743 polymorphism in TSPYL1 (G>A) was associated with increased CYP2C19-mediated metabolism, resulting in lower antidepressant concentrations and poorer clinical response [42].

#### 3.5.2. CYP2D6

Another important gene involved in antidepressant metabolism is **CYP2D6** [19,24,29,31,33].

CYP2D6 polymorphisms were significantly associated with plasma concentrations of antidepressants, underlining their pharmacokinetic relevance (Table 4). The **rs3892097 (G>A) polymorphism**, corresponding to the 4 allele (CYP2D6*4), was particularly relevant. It is associated with decreased enzymatic function and consequently higher drug plasma levels, which may compromise both efficacy and safety.

Patients with the **GG genotype** showed a greater reduction in depression severity scores (HAMD) and experienced fewer adverse effects than A allele carriers.

#### 3.5.3. SLC6A4

The **SLC6A4** gene encodes the serotonin transporter (5-HTT) and was significantly associated with antidepressant treatment outcomes in four studies [20,24,25,42], particularly those involving SSRIs.

The short allele (s) of the 5-HTTLPR polymorphism in the promoter region of SLC6A4 was linked to hypomethylation, resulting in altered gene expression. This was associated with a lower clinical response and lower remission rates.

In contrast, individuals carrying the long (l) allele exhibited better treatment response and higher remission probabilities (Table 5).

#### 3.5.4. Combinatorial Pharmacogenetic Testing

Combinatorial pharmacogenetic testing—an approach that simultaneously evaluates multiple genetic variants affecting both pharmacokinetics and pharmacodynamics—was assessed in two studies [33,34]. These tests, including platforms like GeneSight^®^, integrate information from several genes (e.g., CYP2C19, CYP2D6, CYP3A4) to provide clinical decision support on antidepressant selection and dosing.

Shelton et al. [33] and Parikh et al. [34] reported that combinatorial testing significantly predicted serum concentrations of citalopram, escitalopram, and sertraline, outperforming predictions based on individual gene polymorphisms alone. For example, CYP2C19 and CYP2D6 were found to significantly influence SSRI plasma levels, whereas CYP3A4 showed no independent predictive value.

These findings support the growing use of multigene panels in clinical practice, offering improved prediction of drug metabolism and treatment outcomes compared to single-gene assessments.

#### 3.5.5. Other Genetic Variants

In addition to CYP2C19, CYP2D6, and SLC6A4, several other genes and polymorphisms were explored, albeit in fewer studies, with varying degrees of clinical relevance:

- HTR2A: Two studies [20,36] identified associations between variants in this serotonin receptor gene and treatment response or remission. For example, certain SNPs in HTR2A correlated with depression severity and antidepressant efficacy.
- TSPYL1 (rs3828743): One study [42] found that this variant enhanced CYP2C19 expression, resulting in lower escitalopram plasma levels and poorer clinical outcomes.
- ABCB1: This gene encodes P-glycoprotein, involved in blood-brain barrier transport. Some polymorphisms (e.g., rs1045642, rs2032582) were associated with altered antidepressant distribution and treatment response [22,37].
- BDNF: The Val66Met polymorphism (rs6265), studied in the context of ketamine response [32], did not demonstrate a significant association, though other studies suggest it may impact neuroplasticity and SSRI outcomes.
- COMT: Variants in catechol-O-methyltransferase, such as rs4680 (Val158Met), may influence dopamine metabolism and treatment response to bupropion, with Val carriers responding better to higher doses [39].
- ERICH3 and HTR4: In studies [27,28], polymorphisms in these genes were associated with antidepressant efficacy and remission rates.
- Rare variants: Whole-exome sequencing in one study [23] identified rare functional variants across 35 genes with significant associations to treatment remission (FDR < 0.01), highlighting the potential role of polygenic risk profiles.

While these findings suggest a broader genetic landscape influencing antidepressant treatment outcomes, further studies are required to validate these associations and determine their clinical applicability.

## 4. Discussion

This systematic review highlights the significant role of pharmacogenetics in the pharmacotherapy of major depression, particularly in late-life depression. The analysis of 29 selected studies confirms that certain genetic variants can markedly influence clinical response, tolerability, and safety of antidepressant treatment, especially first-line agents such as SSRIs and SNRIs.

The most extensively studied genes have been CYP2C19 and CYP2D6, which encode cytochrome P450 enzymes involved in the metabolism of antidepressants. Results indicate that patients with an ultrarapid metabolizer phenotype may experience suboptimal treatment response due to faster drug clearance, whereas poor metabolizers tend to exhibit increased adverse effects, resulting from higher plasma drug concentrations. These findings are consistent with those reported by Fabbri and Secretti (2020) [45], who emphasize the importance of CYP2D6 and CYP2C19 genotypes in antidepressant prescribing. They describe how different metabolic profiles influence plasma levels and treatment outcomes, both in terms of efficacy and tolerability. Similar to the current study, a clearer relationship between metabolism and clinical response is observed with escitalopram (SSRI), venlafaxine (SNRI), andTCAs.

Subsequent studies, such as the review by Marin and Milosavljevic (2022) [46], also examine cytochrome P450 enzymes, including CYP2D6, CYP2C19, and CYP2B6, highlighting their key role in drug metabolism. Among the genetic variants analyzed, CYP2C19*17 is noted for its association with ultrarapid metabolism, potentially leading to reduced efficacy. Conversely, the CYP2C19*2 variant, considered nonfunctional, is linked to a higher incidence of adverse effects and poorer treatment tolerability. The authors point out that dosing recommendations based on patient phenotype are becoming increasingly common, as metabolic variations alter drug concentrations. They also emphasize the importance of monitoring other influencing factors such as age, diet, comorbidities, and drug interactions.

More recently, the Clinical Pharmacogenetics Implementation Consortium (CPIC) guidelines (2023) have issued specific recommendations for the use of antidepressants, both SSRIs [47] and TCAs [48], based on CYP2C19 genotype. These guidelines analyze the relationship between genetic variants and drug metabolism and classify individuals into different metabolic phenotypes accordingly. For ultrarapid CYP2C19 metabolizers, switching from SSRIs to antidepressants not primarily metabolized by this enzyme is advised. For poor metabolizers, a 50% dose reduction is recommended to avoid adverse effects.

Beyond these genes, the current review also includes studies investigating polymorphisms in genes related to drug pharmacodynamics, such as SLC6A4. Patients carrying the short (s) allele of the 5-HTTLPR polymorphism show reduced transcription of the serotonin transporter gene and, consequently, exhibit poorer treatment response, lower remission rates, and increased adverse effects. This underscores the importance of pharmacodynamic mechanisms, alongside pharmacokinetics, in antidepressant response. These findings are supported by other studies [5,49] which observe reduced response and remission in patients homozygous for the s allele, who may require higher doses.

In addition to the genetic findings, this review highlights a consistent association between certain genotypes and key clinical outcomes such as remission, treatment failure, therapeutic changes, and adverse events. For example, in the study by Squassina et al. [31], CYP2C19 ultrarapid metabolizers had significantly lower improvement in HDRS-21 scores (*p* = 0.026), while poor metabolizers experienced more frequent therapeutic changes due to adverse effects (*p* = 0.038). Similarly, Mahajna et al. [43] showed that CYP2C19 poor and intermediate metabolizers had increased risk of side effects and lower adherence, and that each unit increase in enzyme activity reduced adverse event risk by 27% (OR = 0.73). These findings are in line with those presented in a 2021 review by Shalimova et al., which emphasized that CYP2C19 variants, particularly CYP2C19 *2 and *17, substantially influence plasma drug levels, efficacy, and tolerability of SSRIs [6].

Regarding *CYP2D6*, individuals with the GG genotype (normal activity) experienced better clinical outcomes and fewer side effects than A allele carriers [29]. Although other studies included in this review analyzed *CYP2D6* variants [31,33,34], no consistent associations with clinical response or tolerability were found. These results are supported by a recent review by Jukic et al. (2022), which highlighted that A allele carriers exhibit reduced enzyme activity, resulting in higher plasma concentrations of several antidepressants, including SSRIs and mirtazapine, thereby increasing the likelihood of adverse effects and suboptimal clinical response [46].

The relationship between genetic variants and depression severity or remission was also evidenced by findings involving SLC6A4, ABCB1, and HTR2A. In one study, hypomethylation of SLC6A4 was significantly associated with lower response and remission rates as measured by the HAMD-21 scale (*p* = 0.010) [25]. Likewise, TSPYL1 variants associated with increased CYP2C19 activity led to lower escitalopram levels and worse response (*p* = 0.0012) [42].

Finally, the review considers the role of combinatorial pharmacogenetic testing that integrates multiple variants. This approach is supported by studies like Brown and Joseph (2022) [50], which suggest that pharmacogenetic-guided antidepressant therapy is associated with a 41% increase in the likelihood of achieving remission. In this review, combined polymorphisms in CYP2C19, CYP2D6, SLC6A4, and HTR2A were analyzed regarding treatment response.

Overall, the findings from this review demonstrate that several genetic variants with potential impact on antidepressant treatment response have been identified in the literature. Polymorphisms primarily in CYP2C19, CYP2D6, and SLC6A4, among others, have been studied. These variants may influence both efficacy and tolerability, providing a comprehensive overview of the role of pharmacogenetics in this clinical context.

This systematic review presents several limitations. First, most of the included studies are observational rather than randomized clinical trials, which may affect the level of evidence. Nevertheless, these studies provide valuable information that reflects real-world clinical practice. Second, the analyzed studies are heterogeneous, encompassing different antidepressant drugs, varied populations, diverse response parameters, and various pharmacogenetic tests. This heterogeneity results in limited evidence and restricts the ability to draw clear conclusions. However, it also allows for a broad and realistic overview of the current state of pharmacogenetic research applied to depression. Third, many reviewed studies do not specifically focus on the geriatric population, which may limit the applicability of the results to late-life depression. Still, some studies include elderly subgroups and provide relevant data that can serve as a basis for more targeted research in this population. Fourth, the potential presence of publication bias may have limited the representativeness of the results. Nonetheless, the literature search was conducted systematically and rigorously, applying clear inclusion and exclusion criteria and consulting three different databases to ensure methodological rigor.

## 5. Conclusions

This study provides a comprehensive and integrated perspective on the role of genetic variants and polymorphisms in the treatment of major depressive disorder, including late-life depression, with antidepressant medications. Several genetic variants with clinically relevant impacts have been identified, influencing both treatment efficacy and tolerability, which highlights the need for further high-quality studies. The main polymorphisms involved in antidepressant metabolism are found in the CYP2C19 and CYP2D6 genes. Specifically, the CYP2C19*17 allele is associated with UM, resulting in faster drug clearance and consequently reduced therapeutic response. Conversely, PM, carrying variants such as *3, have an increased risk of drug accumulation and adverse effects. For CYP2D6, PMs experience more adverse effects and poorer tolerability, while ultrarapid metabolizers (due to gene duplications) may fail to achieve adequate therapeutic concentrations. Other genes, such as SLC6A4, have been linked to treatment efficacy; the presence of the short (S) allele in the promoter region (5-HTTLPR) is associated with a lower response to selective serotonin reuptake inhibitors and a higher risk of side effects. Furthermore, combinatorial pharmacogenetic tests demonstrate superior predictive value compared to single-gene studies. These tests, integrating information from multiple variants, have the potential to optimize antidepressant selection and dosing, thereby improving both treatment efficacy and safety.

## Figures and Tables

**Table 1:** Keywords and bibliographic search results.

Database	Search Terms	Results
PubMed	((pharmacogenetics [Medical Subject Headings (MeSH) Terms] OR pharmacogenomics OR “genetic variability”) AND (antidepressives [MeSH Terms] OR antidepressants OR “selective serotonin reuptake inhibitors” OR SSRIs OR “tricyclic antidepressants”)) AND (depressive disorder [MeSH Terms] OR depression OR “major depressive disorder” OR “late-life depression”)	305
Scifinder	(pharmacogenetics OR pharmacogenomics OR “genetic variability”) AND (antidepressives OR antidepressants OR “selective serotonin reuptake inhibitors” OR SSRIs OR “tricyclic antidepressants”) AND (depressive disorder OR depression OR “major depressive disorder” OR “late-life depression”)	285
Cochrane	(pharmacogenetics OR pharmacogenomics OR “genetic variability”) AND (antidepressives OR antidepressants OR “selective serotonin reuptake inhibitors” OR SSRIs OR “tricyclic antidepressants”) AND (depressive disorder OR depression OR “major depressive disorder” OR “late-life depression”)	203

**Table 2:** Selection criteria.

Inclusion Criteria	Exclusion Criteria
Adults diagnosed with late-life depression (>60 years) or major depressive disorder	Patients with other psychiatric or medical conditions
Observational, prospective, or clinical trial designs	Meta-analyses, systematic reviews, case reports, protocols, guidelines, and other non-original research
Use of antidepressant pharmacotherapy	Use of non-pharmacological treatments
Assessment of pharmacogenetic influence on treatment response, pharmacokinetics and/or safety	No evaluation of pharmacogenetic impact on clinical outcomes
Presence of pharmacogenetic data (genotypes or polymorphisms)	Absence of pharmacogenetic data

**Table 3:** Key CYP2C19 polymorphisms and corresponding phenotypes.

Phenotype	Genetic Variant (Polymorphism/SNP/Change)	Genotype/Allele
Ultrarapid metabolizer	CYP2C19*17—c.-806C>T (rs12248560)	*17/*17 or *1/*17
Normal (extensive) metabolizer	CYP2C19*1—wild-type allele	*1/*1
Intermediate metabolizer	CYP2C19*2—c.681G>A (rs4244285)	*1/*2
	CYP2C19*3—c.636G>A (rs4986893)	*1/*3
Poor metabolizer	CYP2C19*2—c.681G>A (rs4244285)	*2/*2

**Table 4:** Main CYP2D6 Polymorphisms and Clinical Relevance.

Phenotype	Genetic Variant (Polymorphism/SNP/Change)	Genotype/Allele
Better treatment response	CYP2D6*1 functional allel	G/G
Poor treatment response	CYP2D6*4—rs3892097 (1846G>A)	G/A, A/A

**Note**: These *SLC6A4* polymorphisms are associated with serotonin transporter expression and may influence SSRI response and remission.

**Table 5:** Main SLC6A4 Polymorphisms and Their Clinical Impact.

Phenotype	Genetic Variant (Polymorphism/SNP/Change)	Genotype/Allele
Poorer treatment response	5-HTTLPR (44-bp insertion/deletion in promoter region)	L/S or S/S
Better treatment response	5-HTTLPR (44-bp insertion/deletion in promoter region)	L/L
Lower treatment efficacy	rs6354 (C>T) in *SLC6A4*	C allele
Lower treatment efficacy	rs12150214 (G>T) in *SLC6A4*	G allele

Note: Variants in *SLC6A4*, particularly the short allele of 5-HTTLPR and certain SNPs, have been associated with reduced SSRI efficacy and lower remission rates in patients with major and late-life depression [20,24,25,42].

## Data Availability

The template data extraction forms and extracted data from included studies are available from the corresponding author upon reasonable request. No additional unpublished materials were used. All data underlying the findings are derived from published articles included in the review and are listed in Supplementary Material S2.

## Supplementary Materials

The following supporting information can be downloaded at: https://www.mdpi.com/article/10.3390/jcm14145102/s1, Supplementary Materials S1: PRISMA Checklist; Supplementary Materials S2: Clinical and Methodological Characteristics of the 29 Studies Included in the Review; Supplementary Materials S3: Newcastle-Ottawa Scale (NOS) scores of the studies included.

## Abbreviations

The following abbreviations are used in this manuscript:

Abbreviation	Description
AD	Antidepressants
b1-AR	Beta-1 Adrenergic Receptor
BDI	Beck Depression Inventory
BNDF	Brain-Derived Neurotrophic Factor
CES-D	Center for Epidemiologic Studies Depression Scale
CGI	Clinical Global Impressions
COMT	Catechol-O-Methyltransferase
EAC	Randomized Controlled Trials (RCTs)
GRS	Genetic Risk Score
GWAS	Genome-Wide Association Study
HAMD-17	Hamilton Depression Rating Scale-17
HDRS-21	Hamilton Depression Rating Scale-21
IM	Intermediate Metabolizer
ISRS	Selective Serotonin Reuptake Inhibitors (SSRIs)
MDD	Major Depressive Disorder
MDE	Major Depressive Episode
NM	Normal Metabolizers
NDRI	Norepinephrine-Dopamine Reuptake Inhibitor
OMS	World Health Organization (WHO)
PM	Poor Metabolizers
QIDS-C	Quick Inventory of Depressive Symptomatology
SNP	Single Nucleotide Polymorphism
SNRI	Serotonin-Norepinephrine Reuptake Inhibitor
TCA	Tricyclic Antidepressants
TLR	Toll-Like Receptor
TSES	Toronto Side Effects Scale
TSPYL	Testis-Specific Y-Encoded-Like Protein
UM	Ultrarapid Metabolizers
