# Supplementary material for: Pharmacogenetic Implications for Antidepressant Therapy in Major Depression: A Systematic Review Covering 2019–2024
Source: J Clin Med. 2025 Jul 18;14(14):5102. doi: 10.3390/jcm14145102 (PMC12295546; doi:10.3390/jcm14145102)
Supplement: Supplementary file 1 [file jcm-14-05102-s001.zip › jcm-3736459-supplementary.pdf]

Supplementary Material S1. PRISMA Checklist

Section and Topic	Item #	Checklist item	Location where item is reported
**TITLE**			
Title	1	Identify the report as a systematic review.	Line 3
**ABSTRACT**			
Abstract	2	See the PRISMA 2020 for Abstracts checklist.	Line 9
**INTRODUCTION**			
Rationale	3	Describe the rationale for the review in the context of existing knowledge.	Line 46
Objectives	4	Provide an explicit statement of the objective(s) or question(s) the review addresses.	Line 64
**METHODS**			
Eligibility criteria	5	Specify the inclusion and exclusion criteria for the review and how studies were grouped for the syntheses.	Line 98
Information sources	6	Specify all databases, registers, websites, organisations, reference lists and other sources searched or consulted to identify studies. Specify the date when each source was last searched or consulted.	Line 75
Search strategy	7	Present the full search strategies for all databases, registers and websites, including any filters and limits used.	Line 76
Selection process	8	Specify the methods used to decide whether a study met the inclusion criteria of the review, including how many reviewers screened each record and each report retrieved, whether they worked independently, and if applicable, details of automation tools used in the process.	Line 92
Data collection process	9	Specify the methods used to collect data from reports, including how many reviewers collected data from each report, whether they worked independently, any processes for obtaining or confirming data from study investigators, and if applicable, details of automation tools used in the process.	Line 105
Data items	10a	List and define all outcomes for which data were sought. Specify whether all results that were compatible with each outcome domain in each study were sought (e.g. for all measures, time points, analyses), and if not, the methods used to decide which results to collect.	Line 110
	10b	List and define all other variables for which data were sought (e.g. participant and intervention characteristics, funding sources). Describe any assumptions made about any missing or unclear information.	Line 106
Study risk of bias assessment	11	Specify the methods used to assess risk of bias in the included studies, including details of the tool(s) used, how many reviewers assessed each study and whether they worked independently, and if applicable, details of automation tools used in the process.	Line 128
Effect measures	12	Specify for each outcome the effect measure(s) (e.g. risk ratio, mean difference) used in the synthesis or presentation of results.	Not available
Synthesis methods	13a	Describe the processes used to decide which studies were eligible for each synthesis (e.g. tabulating the study intervention characteristics and comparing against the planned groups for each synthesis (item #5)).	Line 92
	13b	Describe any methods required to prepare the data for presentation or synthesis, such as handling of missing summary statistics, or data conversions.	Line 128
	13c	Describe any methods used to tabulate or visually display results of individual studies and syntheses.	Line 116
	13d	Describe any methods used to synthesize results and provide a rationale for the choice(s). If meta-analysis was performed, describe the model(s), method(s) to identify the presence and extent of statistical heterogeneity, and software package(s) used.	Line 113
	13e	Describe any methods used to explore possible causes of heterogeneity among study results (e.g. subgroup analysis, meta-regression).	Line 136

Section and Topic	Item #	Checklist item	Location where item is reported
	13f	Describe any sensitivity analyses conducted to assess robustness of the synthesized results.	Not available
Reporting bias assessment	14	Describe any methods used to assess risk of bias due to missing results in a synthesis (arising from reporting biases).	Line 160
Certainty assessment	15	Describe any methods used to assess certainty (or confidence) in the body of evidence for an outcome.	Line 120
**RESULTS**			
Study selection	16a	Describe the results of the search and selection process, from the number of records identified in the search to the number of studies included in the review, ideally using a flow diagram.	Line 142
	16b	Cite studies that might appear to meet the inclusion criteria, but which were excluded, and explain why they were excluded.	Line 144
Study characteristics	17	Cite each included study and present its characteristics.	Line 182
Risk of bias in studies	18	Present assessments of risk of bias for each included study.	Line 241
Results of individual studies	19	For all outcomes, present, for each study: (a) summary statistics for each group (where appropriate) and (b) an effect estimate and its precision (e.g. confidence/credible interval), ideally using structured tables or plots.	Suppl. Material 2
Results of syntheses	20a	For each synthesis, briefly summarise the characteristics and risk of bias among contributing studies.	Line 241
	20b	Present results of all statistical syntheses conducted. If meta-analysis was done, present for each the summary estimate and its precision (e.g. confidence/credible interval) and measures of statistical heterogeneity. If comparing groups, describe the direction of the effect.	Not available
	20c	Present results of all investigations of possible causes of heterogeneity among study results.	Not available
	20d	Present results of all sensitivity analyses conducted to assess the robustness of the synthesized results.	Not available
Reporting biases	21	Present assessments of risk of bias due to missing results (arising from reporting biases) for each synthesis assessed.	Not available
Certainty of evidence	22	Present assessments of certainty (or confidence) in the body of evidence for each outcome assessed.	Line 185
**DISCUSSION**			
Discussion	23a	Provide a general interpretation of the results in the context of other evidence.	Line 339
	23b	Discuss any limitations of the evidence included in the review.	Line 398
	23c	Discuss any limitations of the review processes used.	Line 387
	23d	Discuss implications of the results for practice, policy, and future research.	Line 382
**OTHER INFORMATION**			
Registration and protocol	24a	Provide registration information for the review, including register name and registration number, or state that the review was not registered.	Line 104
	24b	Indicate where the review protocol can be accessed, or state that a protocol was not prepared.	Not available
	24c	Describe and explain any amendments to information provided at registration or in the protocol.	Not

Section and Topic	Item #	Checklist item	Location where item is reported
			available
Support	25	Describe sources of financial or non-financial support for the review, and the role of the funders or sponsors in the review.	Line 487
Competing interests	26	Declare any competing interests of review authors.	Line 479
Availability of data, code and other materials	27	Report which of the following are publicly available and where they can be found: template data collection forms; data extracted from included studies; data used for all analyses; analytic code; any other materials used in the review.	Line 482

Supplementary Material S2. Clinical and Methodological Characteristics of the 29 Studies Included in the Review

First author	Year of publication	Study type, GRADE evidence	Treatment	Population	Genetic study	Clinical variables assessed	Results related to the study
A. Squassina (31)	2025	Retrospective study. Low evidence. ⊕⊕⊖⊖	SSRIs (46%), SNRIs (36%), TCAs (9%) and others (7%)	156 patients from southern Italy (Sardinia). Caucasian	Genotyping of CYP2D6 and CYP2C19	Number of therapeutic regimens, changes due to adverse effects or lack of response. Response and remission measured using Hamilton Depression Rating Scale-21 (HDRS-21).	CYP2C19 UMs showed significantly less clinical improvement compared to Normal Metabolizer (NMs) (p=0.026) via HDRS-21. Poor Metabolizer (PM)s had more treatment changes due to adverse effects than NMs (p=0.038). No significant differences were observed according to CYP2D6 genotype. The study suggests CYP2C19 has more predictive value than CYP2D6 for antidepressants (AD) response.
S. H. Kanders (16)	2020	Prospective study. Low evidence. ⊕⊕⊖⊖	SSRIs, SNRIs, TCAs, and other combined ADs	150 patients from the Swiss cohort. Caucasian	3 SNPs: rs12248560 (CYP2C19), rs878567 (HTR1A), rs17710780 (ARHGEF37)	Occurrence of major depressive episodes (MDE) and depression severity measured with Center for Epidemiologic Studies Depression Scale (CES-D) and ln(CES-D). Treatment adherence (regular vs. occasional) evaluated.	A Genetic Risk Score (GRS) including the 3 SNPs was significantly associated with lnCES-D (p=0.001) and MDE occurrence (p=0.02). Carriers of the G allele in HTR1A (rs878567) had lower lnCES-D scores (p=0.02). The C allele of CYP2C19 (rs12248560) was associated with regular treatment (p=0.03), whereas TT/Ultrarapid Metabolizers (UM) carriers had higher treatment failure.
J. Wang (17)	2020	Prospective study. Low evidence. ⊕⊕⊖⊖	Escitalopram, citalopram, sertraline (SSRIs)	438 East Asian patients with first major	3 TLR4 SNPs: rs1927911, rs11536889, rs7873784	Antidepressant efficacy measured with HAMD-17 and five associated MDD severity factors	No significant associations between toll-like receptor (TLR4) polymorphisms and treatment response, efficacy

Supplementary Material S2. Clinical and Methodological Characteristics of the 29 Studies Included in the Review

First author	Year of publication	Study type, GRADE evidence	Treatment	Population	Genetic study	Clinical variables assessed	Results related to the study
				depressive disorder (MDD)		(anxiety, cognitive symptoms, weight loss, insomnia, and retardation symptoms).	($p>0.11$), or depression remission ($p>0.45$). These polymorphisms were associated with MDD symptom presence.
N. Firouzabadi (18)	2020	Prospective study. Low evidence. ⊕⊕⊖⊖	Fluoxetine (SSRI)	101 Iranian patients without prior antidepressant treatment. Caucasian	G1165C (Arg389Gly) polymorphism in beta-1 adrenergic receptor (β1AR) receptor gene	Reduction in HAMD-21 scale score before and after treatment	No significant genotypic ($p=0.905$) or allelic ($p=0.568$) associations between G1165C polymorphism and HAMD-21 score reduction, nor with AD treatment response.
N. B. Rodrigues (32)	2024	Retrospective study. Low evidence. ⊕⊕⊖⊖	Intravenous ketamine	85 patients who received at least 4 ketamine infusions. Ethnicity not specified.	Brain-Derived Neurotrophic Factor (BDNF) Val66Met and CYP2B6 polymorphisms	Self-reported depressive symptoms via Quick Inventory of Depressive Symptomatology – Clinician Rated (QIDS-SR16).	No significant association between either BDNF Val66Met ($p=0.47$) or CYP2B6 ($p=0.97$) and treatment response to ketamine.
R. C. Shelton (33)	2020	Retrospective study. Low evidence. ⊕⊕⊖⊖	Citalopram and Escitalopram (SSRIs)	191 patients (96 on citalopram, 95 on escitalopram). Ethnicity not specified.	CYP2C19, CYP2D6, CYP3A4 genes and combinatorial pharmacogenetic testing (GeneSight®)	SSRI blood concentrations, dose-response relationship	Pharmacogenetic testing significantly predicted SSRI blood levels ($p=0.00003$). Significant effects for CYP2C19 ($p=0.01$) and CYP2D6 ($p=0.03$); CYP3A4 was not significant.
S. V. Parikh (34)	2020	Post-hoc analysis of the GUIDED study. Low evidence. ⊕⊕⊖⊖	Sertraline (SSRIs)	124 patients treated with sertraline. Ethnicity not specified.	CYP2C19, CYP2B6, CYP3A4 genes and combinatorial pharmacogenetic testing (GeneSight®)	Sertraline blood concentrations, dose-response relationship	Pharmacogenetic testing significantly predicted sertraline levels ($p=6.3\times10^{-8}$). Significant influence from CYP2C19 ($p=2.7\times10^{-6}$) and CYP2B6 ($p=0.03$). CYP3A4 was not significant ($p=0.8$).

Supplementary Material S2. Clinical and Methodological Characteristics of the 29 Studies Included in the Review

First author	Year of publication	Study type, GRADE evidence	Treatment	Population	Genetic study	Clinical variables assessed	Results related to the study
D. Jokovic (35)	2022	Retrospective study. Moderate evidence. ⊕⊕⊕⊖	Escitalopram, citalopram, sertraline (SSRIs), venlafaxine, fluoxetine, mirtazapine, etc.	102 hospitalized patients. Caucasian	CYP2C19 polymorphisms and classification as PM, NM, rapid metabolizer (RM)	Efficacy via HAMD, Beck Depression Inventory (BDI)-IA, Clinical Global Impressions (CGI). Tolerability via Toronto Side Effects Scale (TSES), CGI-E.	PMs had 36-43% lower HAMD scores ($p<0.0001$). RMs had 75% less frequent clinical response ($p<0.01$). PMs had more adverse effects ($p<0.05$). No efficacy/tolerability differences between NMs and RMs.
S. Qin (42)	2020	Observational retrospective study. Moderate evidence. ⊕⊕⊕⊖	SSRIs: Escitalopram and Citalopram	Patients with MDD in clinical trials. Ethnicity not specified.	Testis-Specific Y-Encoded-Like Protein (TSPYL)1 (rs3828743), TSPYL2, TSPYL4 polymorphisms; influence on CYP2C19 and SLC6A4 expression	Clinical efficacy via QIDS-C, HAMD; SSRI response; plasma concentrations of SSRI and metabolite (S-DCT)	TSPYL1 (rs3828743) associated with increased CYP2C19 metabolism, lower escitalopram levels, and worse response ($p=0.0012$). Wild-type (G) associated with higher AD levels ($p=0.0497$) and lower metabolite/drug ratio ($p=0.0289$). TSPYLs modulate SERT (SLC6A4), decreasing synaptic serotonin and increasing depressive symptoms.
M. Mahajna (43)	2023	Retrospective cohort study. Moderate evidence. ⊕⊕⊕⊖	SSRIs: Escitalopram and Citalopram	283 Israeli patients on monotherapy. Ethnicity not specified.	CYP2C19 polymorphisms classified as PM, IM, NM, RM, UM	Adverse effects, treatment adherence, therapeutic changes	PM/IM had higher adverse effect risk than RM/UM ($p<0.05$). Each unit increase in CYP2C19 activity reduced adverse effect probability by 27% (OR=0.73). Patients with low adherence and frequent changes were more likely PMs.
M. S. Zastrozhin (30)	2021	Observational prospective study. Moderate	Citalopram (SSRI)	130 male patients with comorbid alcohol use disorder.	CYP2C19 681G>A polymorphism (rs4244285)	Steady-state citalopram concentration, efficacy using HAMD, safety using UKU	GA genotype was associated with higher steady-state citalopram concentration ($p<0.001$), lower efficacy

Supplementary Material S2. Clinical and Methodological Characteristics of the 29 Studies Included in the Review

First author	Year of publication	Study type, GRADE evidence	Treatment	Population	Genetic study	Clinical variables assessed	Results related to the study
		evidence. ⊕⊕⊕⊖		Ethnicity not specified.			(higher HAMD, p<0.001), and reduced safety (more adverse events, higher UKU, p<0.001).
C.F. Kao (36)	2020	Retrospective observational study. Moderate evidence. ⊕⊕⊕⊖	Escitalopram, Citalopram (SSRIs) Paroxetine, Fluoxetine, Sertraline (SSRI), venlafaxine.	455 patients from Taiwan. East Asians.	288 polymorphisms of the *HTR2A gene*	The response to HRSD treatment and remission of depression.	None of the polymorphisms has evidence for susceptibility to MDD. Thirteen out of 14 SNPs are associated with remission of depression (p< 5x10-3) and 1 out of 8 SNPs are associated with AD response.
M.S. Zastrozhin (19)	2020	Prospective observational study. Moderate evidence. ⊕⊕⊕⊖	Duloxetine (SNRI)	118 male patients. Ethnicity not specified.	184G>A *CYP2D6* polymorphism (rs3892097).	The concentration/dose ratio of Duloxetine at steady state, treatment efficacy with the HAMD scale, and safety with UKU.	The GA genotype of *CYP2D6* is associated with a higher concentration/dose ratio (p<0.001), therefore, with lower metabolism. It is also associated with lower efficacy with a higher HAMD value (p<0.001), and lower safety with a greater number of adverse effects and a higher UKU value (p=0.007).
A. Jelen (37)	2023	Retrospective observational study. Moderate evidence. ⊕⊕⊕⊖	SSRIs, venlafaxine, agomelatine and combinations of AD.	102 patients. Caucasians	Polymorphisms of the *ABCB1 gene*: T-129C, C1236T, G2677T/A and C3435T.	The effectiveness of the treatment and the severity of symptoms using the HDRS scale.	The combinations of SNPs 1236T, 2677T/A and 3435T were associated with greater symptom severity and a significantly lower response to treatment (p<0.05). T-129C did not show a significant relationship with the response to AD (p=0.3176).

Supplementary Material S2. Clinical and Methodological Characteristics of the 29 Studies Included in the Review

First author	Year of publication	Study type, GRADE evidence	Treatment	Population	Genetic study	Clinical variables assessed	Results related to the study
Yan Bi (20)	2021	Prospective observational study. Moderate evidence. ⊕⊕⊕⊖	ISRS (fluoxetina, citalopram, sertralina, paroxetina), SNRI (venlafaxina, duloxetina), TCA i NaSSA (mirtazapina).	610 Chinese patients. East Asia	127 SNPs of different genes: *SLC6A4* (rs6354 and rs12150214), *MAOA* (rs6323, rs1137070), *HTR2A*, *ABCB1*.	The effectiveness of the treatment and remission of depression with the HAMD scale.	*SLC6A4* gene variants (rs6354 C-allele carriers and rs12150214 G-allele carriers) were associated with worse efficacy in fluoxetine ($p<0.07$) and *MAOA* (rs6323 and rs1137070) treatment in women ($p<0.07$).
M. S. Zastrozhin (21)	2022	Prospective observational study. Moderate evidence. ⊕⊕⊕⊖	Escitalopram (ISRS)	267 male patients Ethnicity not specified.	Polymorphism *CYP2C19*17* (806C>T)	Steady-state Escitalopram concentration, treatment efficacy using the HAMD and HADS scale, and safety using UKU.	Ultrarapid metabolisers *of CYP2C19*17* showed a better clinical response with a significantly greater reduction in the HAMD scale ($p<0.001$) and fewer adverse effects measured with the UKU scale ($p<0.001$).<br>The CYP2C19*17 variant did not show a significant association with the relationship between plasma concentration and dose of escitalopram.
Mr. Calabro (38)	2022	Retrospective observational study. Moderate evidence. ⊕⊕⊕⊖	SSRIs: Citalopram, escitalopram and sertraline. Also SNRI, ED and other psychotropic drugs in group 2.	1239 patients. Ethnicity not specified.	CYP2C19 polymorphisms and classification according to their metabolising character: PM, IM, NM, RM, UM	Treatment outcomes, treatment changes and side effects using the UKU scale.	The CYP2C19 PMs had a greater number of adverse effects because they have a higher plasma concentration of the drug ($p<0.05$) compared to the other metabolising traits.<br>UM compared to ME/MI had less response to treatment and more changes in it ($p<0.05$).

Supplementary Material S2. Clinical and Methodological Characteristics of the 29 Studies Included in the Review

First author	Year of publication	Study type, GRADE evidence	Treatment	Population	Genetic study	Clinical variables assessed	Results related to the study
M. Simoonsa (22)	2020	Prospective observational study Moderate evidence. ⊕⊕⊕⊖	Paroxetine (ISRS)	81 patients. Ethnicity not specified.	*ABCB1* gene polymorphisms (code-for P-glycoprotein): rs1045642, rs1128503, rs2032582 and rs2235040.	Serum paroxetine concentration, serotonin transporter (SERT) occupancy, and clinical response to treatment with the HDRS17 scale.	The polymorphisms rs1128503 and rs2032582 significantly modified the relationship between serum paroxetine concentration and serotonin transporter occupancy. ABCB1 gene polymorphisms were not significantly associated with clinical response to treatment ($p>0.05$).
M.L. Wong (23)	2021	Prospective randomized and double-blind study. Moderate evidence. ⊕⊕⊕⊖	Fluoxetina (ISRS) i Desipramina (TCA).	65 Mexican-American patients. Ethnicity not specified.	Whole exome genotype and determination of rare functional variants.	Clinical efficacy and response to treatment with the HAM-D scale.	35 genes with rare variants that are significantly associated ($FDR<0.01$) with remission of depression and treatment efficacy.
D. Jarcusková (24)	2024	Prospective observational study Low evidence ⊕⊕⊖⊖	Escitalopram (SSRI) and the use of benzodiazepines temporarily. Secondary treatment with other ADs.	88 Slovak patients. Caucasians	Genetic modifications of: *5-HTTLPR (*encoded by transporter SLC6A4), HTR2A rs9316233, *BDNF* rs962369, *CYP2C19* and *CYP2D6*.	Remission using the HAMD-21 and response to treatment (reduction >50% HAMD-21).	A significant association was found between the genes studied and clinical response. Carriers of the short allele (S) of the *5-HTTLPR* gene had a significantly lower response than carriers of the long allele (L) ($p=0.01$).
M. A. Schiele (25)	2021	Observational study Moderate evidence. ⊕⊕⊕⊖	AD: ISRS, SNRI, TCA, NaSSA and others.	236 hospitalized patients with a subgroup of 110 patients only treated with SSRIs and SNRIs.	SLC6A4 *gene promoter methylation* (serotonin transporter gene) at 9 CpG sites	Remission using the HAMD-21 and response to treatment (reduction >50% HAMD-21).	There was a significant association between hypomethylation of the *SLC6A4 gene* and lower response in dimensional HAM-D ($p=0.015$), which implies relative reductions in HAM-D scores, in

Supplementary Material S2. Clinical and Methodological Characteristics of the 29 Studies Included in the Review

First author	Year of publication	Study type, GRADE evidence	Treatment	Population	Genetic study	Clinical variables assessed	Results related to the study
				All of Caucasian origin.			categorical treatment (p=0.021), HAM-D reduction >50%, and in a lower remission (p=0.010). The effect was also significant in the subgroup of 110 patients.
K. Chapell (26)	2022	Prospective observational study (cohort) Moderate evidence. ⊕⊕⊕⊖	AD: SSRI (40%), SNRI (41%), TCA and others. Always monotherapy.	388 patients. Caucasians (91%), Africans and mixed origin.	Complete *ARRB1* sequencing: rare and common variants such as *rs553664* and *rs53685*.	Remission of depression using the HDRS-17 scale and response to treatment (reduction >50%).	The sum of the different rare variants significantly negatively influenced the changes in HDRS-21 score (p=0.0033), response (p=0.016) and remission (p=0.022).<br>The most common variants with significantly lower remission rates (higher HDRS values) were: rs553664AA compared to AG (p=0.014) and rs53685 GG compared to AG (p=0.0018)
J. Fawyer (39)	2020	Retrospective observational study Moderate evidence. ⊕⊕⊕⊖	Bupropion (Norepinephrine-Dopamine Reuptake Inhibitor (NDRI))	241 outpatients, most of them Caucasian (85.9%)	Genotip Catechol-O-Methyltransferase (*COMT)* (rs4880) with/with, with/fall o fall/trap	The PHQ-9 score (patient health questionnaire) before and after AD treatment.	Bupropion in Val carriers (Val/Val or Met/Val) at high doses was more effective than at low doses (p=0.01). There was a better response to treatment with lower PHQ-9 scores in Val carriers compared to the Met/Met genotype (p=0.04).
W. L. E. Wong (40)	2023	Retrospective observational study Moderate evidence. ⊕⊕⊕⊖	Citalopram, escitalopram i sertralina (ISRS)	33094 patients Ethnicity not specified.	Metabolizing character of *CYP2C19*: PM, IM, NM and MRI	Medication changes, duration of treatment, discontinuation and the appearance of side effects.	PMs with respect to NM in patients treated with escitalopram presented more adverse effects (p=0.041) and more changes in treatment (p=0.015), while RMs with

Supplementary Material S2. Clinical and Methodological Characteristics of the 29 Studies Included in the Review

First author	Year of publication	Study type, GRADE evidence	Treatment	Population	Genetic study	Clinical variables assessed	Results related to the study
							respect to NM presented less probability of change (p=0.036). In citalopram treatment, PM and MI had a shorter duration of treatment compared to NM (p=0.045) No significant associations were observed between the metabolizing character of *CYP2C19* and sertraline.
K. Chappell (27)	2022	Prospective observational study. Moderate evidence ⊕⊕⊕⊖	SSRIs (escitalopram, citalopram and paroxetine), SNRI (venlafaxine) and others (mirtazapine)	377 patients from the METADAP cohort. Caucasians.	RS11580409 (A>C) *ERICH3* polymorphism and genotypes (CC, AA, AC)	Remission of depression with the HDRS-17 scale and response to treatment (reduction >50%). It also measured plasma serotonin concentrations.	Patients with CC genotype showed a greater reduction in the HDRS scale (p=0.019) and an increase in the response rate (p=0.039) compared to carriers of allele A. No significant relationship was found with plasma serotonin concentrations (p=0.26).
V. Poinsignon (28)	2022	Prospective observational study Moderate evidence. ⊕⊕⊕⊖	SSRIs (escitalopram, citalopram and paroxetine), SNRI (venlafaxine) and others (mirtazapine)	492 patients from the METDAP cohort. Mostly Caucasians.	Genotip receptor *HTR4* rs1345697 (GG, AG, AA)	Treatment response and remission rate with the HDRS-17 scale (reduction >50%)	Patients with the GG genotype had a lower remission rate (p=0.05) and less improvement in treatment (p=0.04) than carriers of allele A (AA/AG). A better prognosis was observed with treatment in carriers of the A allele.
Zastrozhin (29)	2020	Prospective observational study Moderate evidence.	Mirtazapina	192 male patients with alcohol use disorder. Ethnicity not specified.	Polymorphism (1846G>A) *CYP2D6*4* rs3892097 and microRNA-hsa-miR-370-3p concentration	Steady-state mirtazapine and microRNA concentration, treatment efficacy with the HAMD	Patients with the GG by CYP2D64 genotype showed better treatment efficacy with a greater reduction in HAMD

Supplementary Material S2. Clinical and Methodological Characteristics of the 29 Studies Included in the Review

First author	Year of publication	Study type, GRADE evidence	Treatment	Population	Genetic study	Clinical variables assessed	Results related to the study
		⊕⊕⊕⊖				and HADS scale, and safety with UKU.	score (p<0.001) and fewer adverse effects (p<0.001).<br>The concentration of microRNAs had a statistically significant relationship with CYP2D64 expression (p<0.001) but not in clinical response (p=0.460).
M-H. Cheng (44)	2021	Clinical trial.<br>High evidence.<br>⊕⊕⊕⊕	Ketamine	65 patients.<br>Ethnicity not specified.	684616 SNPs of 12 genes involved in the ketamine response: *BDNF, CYP2B6, NTRK2, MTOR* and *NDMAR* genes.	Evaluation of the response to treatment with the HDRS-17 and MADRS scales (reduction >50%)	Polymorphisms in *BDNF* (rs2049048), NTRK2 (rs1027777, rs10868590, rs77918527) and *NDMAR genes* (*GRIN2A. GRIN2B and GRIN2C*) showed a significant association with the response to AD treatment due to the reduction of the values of the two scales (p<0.01) and an improvement in depression.
JH. Park (41)	2021	Moderate evidence.<br>⊕⊕⊕⊖	ISRS (escitalopram, citalopram i sertralina)	100 patients from the discovery cohort and 553 from the replication cohort.<br>Ethnicity not specified.	Variant *KRTAP1-1* rs3213755.	HDRS-17 scale, which assesses remission of depression (HDRS-<8) and response to treatment (reduction >50%).	Allele A of the *KRTAP1-1* gene (rs3213755) was associated with a lower probability of remission in the discovery cohort (p=0.0184) and in the replication cohort (p=0.00269).<br>The rs3213755 variant was associated with a reduction in *KRTAP1-1 expression* (p=0.045).

Supplementary Material S3. Newcastle-Ottawa Scale (NOS) scores of the studies included.

Study	Selection (S) 1	S2	S3	S4	Comparability (C)	Exposure (E)1	E2	E3
A. Squassina 2025	*	-	*	*	**	*	*	-
S. H. Kanders 2020	*	-	*	-	**	*	*	-
J. Wang 2020	*	-	*	*	-	*	*	-
N. Firouzabadi 2020	*	-	*	*	*	*	*	-
N. B.Rodrigues 2024	*	-	*	*	**	*	*	-
R. C. Shelton 2020	*	-	*	*	**	*	*	-
S. V. Parikh 2020	*	-	*	*	**	*	*	-
D. Jokovic 2022	*	-	*	*	**	*	*	-
S. Qin 2020	*	-	*	*	*	*	*	-
M. Mahajna 2023	*	*	*	*	**	*	*	-
M. S. Zastrozhin 2021	-	-	*	*	*	*	*	-
C.F. Kao 2020	*	*	*	*	*	*	*	-
M.S. Zastrozhin 2020	-	-	*	*	*	*	*	
A. Jelen 2023	*	-	*	*	*	*	*	-
Yan Bi 2021	*	-	*	*	**	*	*	-
M. S. Zastrozhin 2022	*	-	*	*	-	*	*	-
M. Calabro 2022	*	-	*	*	*	*	*	-
M. Simoonsa 2020	*	-	*	*	**	*	*	-
M.L. Wong 2021	*	-	*	*	-	*	*	*
D. Jarcusková 2024	*	-	*	*	*	*	*	-
M. A. Schiele 2021	*	-	*	*	-	*	*	-

K. Chapell 2022	*	-	*	*	**	*	*	*
J. Fawyer 2020	*	-	*	*	*	*	*	-
W. L. E. Wong 2023	*	*	*	*	*	*	*	-
K. Chappell 2022	*	*	*	*	**	*	*	*
V. Poinsignon 2022	*	*	*	*	**	*	*	-
Zastrozhin 2020	-	-	*	*	-	*	*	-
M-H. Cheng 2021	*	-	*	*	*	*	*	-
JH. Park 2021	*	-	*	*	*	*	*	-
